# Physical activity to reduce PCSK9 levels

**DOI:** 10.3389/fcvm.2022.988698

**Published:** 2022-08-25

**Authors:** Amedeo Tirandi, Fabrizio Montecucco, Luca Liberale

**Affiliations:** ^1^First Clinic of Internal Medicine, Department of Internal Medicine, University of Genoa, Genoa, Italy; ^2^IRCCS Ospedale Policlinico San Martino Genoa - Italian Cardiovascular Network, Genoa, Italy

**Keywords:** proprotein convertase subtilisin/kexin type 9, physical activity, exercise, inflammation, cardiovascular

## Abstract

The amount of physical activity (PA) people practice everyday has been reducing in the last decades. Sedentary subjects tend to have an impaired lipid plasma profile with a higher risk of atherosclerosis and related cardio- and cerebrovascular events. Regular PA helps in both primary and secondary cardiovascular prevention because of its beneficial effect on the whole metabolism. Several studies reported lower levels of plasma lipids in trained subjects, but the precise mechanisms by which PA modulates lipoproteins remain only partially described. Thereupon, proprotein convertase subtilisin/kexin type 9 (PCSK9) is a serin protease whose main function is to reduce the amount of low-density lipoprotein cholesterol (LDL-C) receptors, with the direct consequence of reducing LDL-C uptake by the liver and increasing its circulating pool. Accordingly, recently developed PCSK9 inhibitors improved cardiovascular prevention and are increasingly used to reach LDL-C goals in patients at high CV risk. Whether PA can modulate the levels of PCSK9 remains partially explored. Recent studies suggest PA as a negative modulator of such a deleterious CV mediator. Yet the level of evidence is limited. The aim of this review is to summarize the recent reports concerning the regulatory role of PA on PCSK9 plasma levels, highlighting the beneficial role of regular exercise on the prevention of atherosclerosis and overall CV health.

## Introduction

Regular exercise has been recommended to improve both quality and quantity of life in different clinical settings. The importance of fitness for a healthier life is particularly important nowadays, since the modern society tends to underestimate the time spent sitting in front of device screens, with almost two billion of physically inactive subjects worldwide (1). From being nomadic hunter gatherers, we became settled agriculturalists. Nowadays, we tend to spend even less time outdoor with many works are now available online and can be done at home using an internet connection. In this context, the recent severe acute respiratory syndrome coronavirus 2 (SARS-CoV2) pandemic did not help. Epidemiological data report that the total amount of physical activity (PA) has progressively reduced in the last decades (2), meanwhile the number of obese subjects almost tripled worldwide (3). Obesity is a well-known cardiovascular risk factor and is associated with a high number of comorbidities involving most organs of our bodies.

However, physical activity might be aerobic or anaerobic. The aerobic training consists in exercises involving large group of muscles in a rhythmic and dynamic manner for a long time, while the anaerobic training in a more intense activity, exerted in a shorted time, and involving a restricted group of muscles (4). Even though, the aerobic physical exercise has always been regarded as healthier and therefore preferable, recent evidence shows that both aerobic and anaerobic physical activity may have beneficial effect on the cardiovascular system (4). Regular PA has protective effects against cardiovascular diseases and all-cause mortality (5) and atherosclerosis is negatively modulated by regular exercise (6). Even slight increase of daily amount of PA, in the magnitude of 30 min of light- to vigorous-intensity PA per day can significantly improve cardiovascular health (7). Greater results are obtained with regular high-intensity exercise training (8).

Our body has a greater ability to face PA and starvation periods rather than an excess of caloric intake and sedentarism. We have many hormonal pathways that can mobilize depots and increase circulating glucose and lipid levels, instead the machinery to reduce their levels is rather limited. As a result, the excess of caloric intake associated with low exercise training favor the development of metabolism impairment. The direct consequence is the slowly progression toward the so-called “X syndrome,” better known as the metabolic syndrome. This syndrome is associated with several cardiovascular diseases (9) and sudden cardiac death (10). CV prevention therefore finds a cornerstone in strategies aiming at regulating lipid levels by reducing low-density lipoprotein cholesterol (LDL-C) and increasing high-density lipoprotein cholesterol (HDL-C). PA was shown to have beneficial effects on lipid profile (with sex-related differences), especially when coupled with better dietary habits (11, 12). When this is not enough to reach the target lipoprotein levels suggested by the guidelines, pharmacological approaches including statins and PCSK9 inhibitors are suggested. In consideration of the recent paradigm for cholesterol levels “the lower the better,” the use of proprotein convertase subtilisin/kexin type 9 (PCSK9) inhibitors drugs such as alirocumab and evolocumab to reduce circulating LDL-C in patient at intermediate to very high risk is getting more and more common in the clinical practice.

PCSK9 is a serine protease whose main role is to favorite the catabolism of LDL-C receptor on the hepatocyte surface thereby reducing LDL-C internalization and increasing its circulating pool (13, 14). Reducing the PCSK9 plasma levels therefore leads to reduced circulating cholesterol levels with direct inhibitory effects on the atherosclerotic process. Yet, PCSK9 recently showed pleiotropic, non-LDL-C-mediated, effects that are nowadays known to mediate some of the anti-atherosclerotic effects of PCSK9 inhibitors (i.e., through inflammation) (15, 16). In fact, low grade inflammation is associated with PCSK9 transcription, especially in metabolic syndrome patients (17). Furthermore, inflammation favorite the expression of PCSK9 in the endothelial cells and vascular smooth muscle cells (18).

Whether PCSK9 mediates some of the beneficial effects of PA on plasma lipoproteins remains to be fully investigated. Recent evidence showed that PCSK9 plasma levels are regulated by regular exercise in both animal models and humans, as reported in Table 1. Although, the strength of this association and the possible pathways are not precisely described. The purpose of this review is to highlight the role of exercise training on PCSK9 plasma levels, as a prevention strategy against atherosclerosis. We searched PubMed and Web of Science for the following keywords: “proprotein convertase/subtilisin kexin type,” “PCSK9” in combination with “exercise” and “physical activity.” Additionally, a list of recent pre-clinical and clinical studies concerning this topic is reported as a table and discussed.

**Table 1:** Summary of recent pre-clinical and clinical studies evaluating the effect of exercise training on PCSK9.

**Pre-clinical research**				
**Author**	**Year**	**Model**	**Intervention**	**Findings**
Wen et al. (19)	2013	Mouse	AET on treadmill and different types of diet lasting up to 8 weeks	Treadmill exercise increases hepatic PCSK9 mRNA while reducing circulating PCSK9. Reduced lipid levels in mice fed with high-fat diet
Ngo Sock et al. (20)	2014	Rat	Treadmill AET for 8 weeks	Exercise training has no effect on circulating PCSK9 even if it reestablishes the expression of sterol regulatory element binding protein 2
Farahnak et al. (21)	2018	Rat	AET *via* voluntary wheel running for 6 weeks	Increasing of intestinal LDL-R and PCSK9 transcripts in both intact and ovariectomized animals, indicating a possible role in the trans-intestinal cholesterol excretion
Li et al. (22)	2020	Rat	AET on treadmill for 8 weeks	Increase of hepatic LDL-R; inhibition of neointimal formation *via* PCSK9 and LOX-1 reduction
Wolf et al. (23)	2021	Rat	AET *via* voluntary wheel running for 10 months	Exercise favors the expression of PCSK9 in the muscles of normotensive rats without affecting circulating pool.
**Clinical**				
**Author**	**Year**	**Population**	**Intervention**	**Findings**
Arsenault et al. (24)	2014	Obese, sedentary men aged between 30 and 65 years old	Moderate AET (160 min/week), more occupational PA, and diet	Modest reduction of PCSK9 levels after 1 year. PCSK9 is slightly associated with insulin resistance but not with LDL-C plasma levels
Kamani et al. (25)	2015	Hospital employees aged more than 18 years old	Use of stairs instead of elevator at workplace for up to 6 months	Reduction of circulating levels of PCSK9 up to 20% at 3rd month but similar levels to the baseline at 6th month
Boyer et al. (26)	2016	Men 39–80 years old undergoing coronary artery bypass graft	150 min/week of PA, and diet program for 1 year	Increment of PCSK9 in relation with fitness and visceral fat mobilization; no LDL-C modification
Sponder at al. (27)	2017	Subjects aged between 30 and 65 years old with at least one cardiovascular risk factor	Moderate-vigorous AET for 8 months, from 75 min/week of high-intensity to 150 min/week of moderate-intensity PA	Reduction of LDL-C levels with increased circulating PCSK9
Makela et al. (28)	2019	Sedentary, pre-diabetic, middle aged patients	AET for 60 min three times in a week for up to 3 months	Reduction of PCSK9 plasma levels even though low intensity PA seems not to influence PCSK9 levels

*AET, aerobic exercise training; LDL-r, low-density lipoprotein cholesterol receptor; LOX-1, low-density lipoprotein receptor-1; mRNA, messenger ribonucleic acid; PCSK9, proprotein convertase subtilisin/kexin type 9*.

## The effect of exercise training on low-density lipoprotein cholesterol

Exercise training consists in a structured and repetitive PA that is practiced regularly. As longtime known, the amount and the quality of exercises and trainings can determine different results ranging from beneficial to even negative results in different settings (29). As a result, one of the major pitfalls in comparing different trials is due to the different type, intensity, and duration of the exercise protocol. Concerning the effect of exercise on cholesterol, several preclinical (30–32) and clinical studies (33–35) found an amelioration of the lipid plasma profile *via* regular exercise, especially aerobic one.

The modulation of circulating LDL-C in trained animals and humans relates to the adaptation of the body toward a higher metabolic state. Accordingly, the effects of exercise can be seen even acutely. Immediately after PA, LDL-C plasma levels decreases (36, 37). After termination, LDL-C levels tend to return to their basal levels, yet with regular exercise they remain lower on the long-term (38). Concerning the abovementioned adaptations, a higher metabolic state associates with a reduction of PCSK9. Consequently, the reduced catabolism of LDL receptor, increases the uptake of circulating LDL thereby reducing circulating cholesterol levels. Accordingly, recent evidence showed that PA increases the amount of hepatic LDL-C receptor (19, 39). Table 1 summarizes the main evidence proving an effect of PA on PCSK9 levels. As most studies agree on a beneficial lowering effect, an augmentation of PCSK9 levels may coexist together with LDL-C reduction in well-trained people (27). Such mixed effect might be due to many limitations in the analysis and interpretation of the studies. Furthermore, the amount of evidence concerning this topic remains quite limited.

### Mediators of PCSK9 modulation in trained subjects

**Figure 1:**
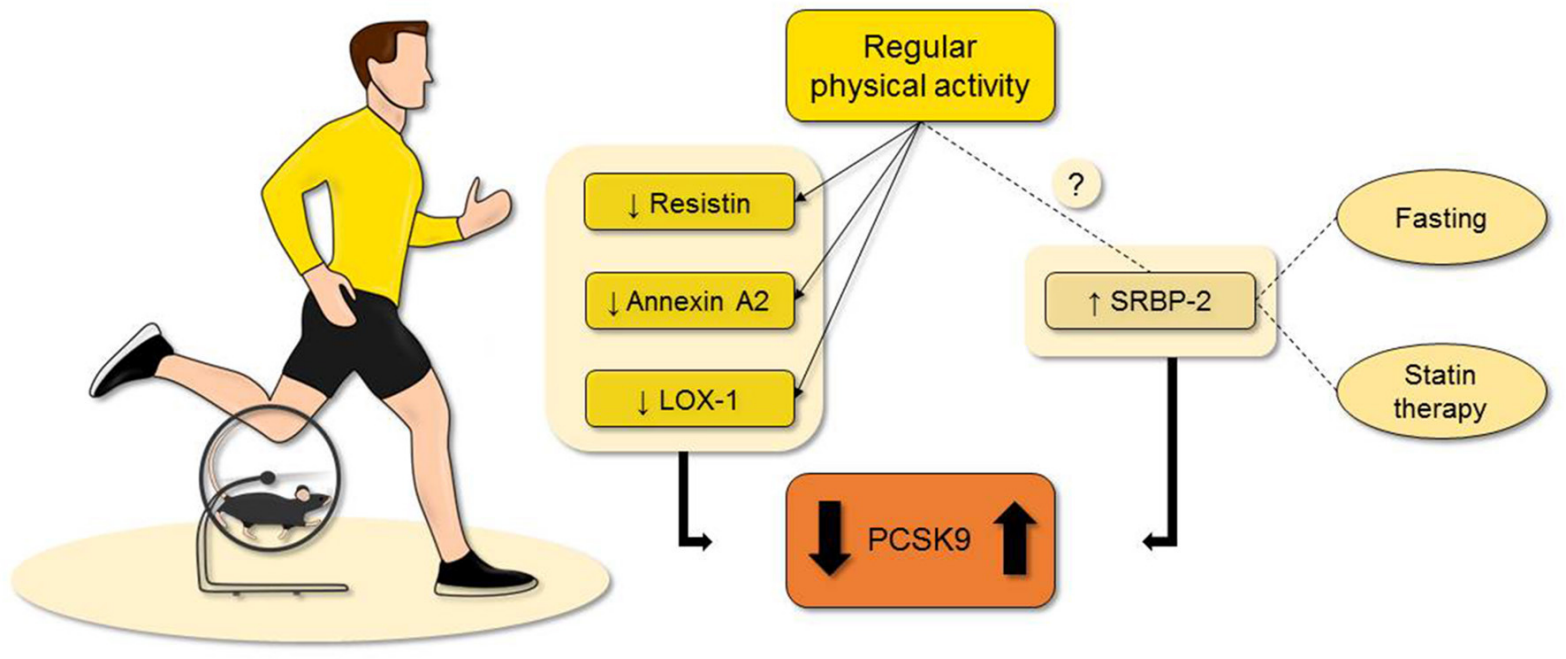
Evidence suggest that regular physical activity can regulate the PCSK9 plasma levels even though few evidence is available at the moment. As possible explanations, the reduction of resistin, annexin A2, and LOX-1 are reported with the most concrete results. Although, PCSK9 can be also augmented in trained subjects. This is probably related to a higher metabolism, a possible undiscovered mechanism, or confounding factors. While it is less known if exercise training affects the plasma levels of SREBP2, both fasting and statin treatment represent two confounding factors when evaluating the possible role of physical activity on PCSK9 levels. LOX-1, lectin-type oxidized low-density lipoprotein receptor-1; Proprotein convertase subtilisin/kexin type 9; SREBP2, sterol regulatory element-binding protein 2.

Trained people tend to show higher amount of lean mass and lower levels of visceral fat (40–42). As known today, visceral fat is not only a storage tissue, but it plays a crucial role in the pathophysiology of different diseases (e.g., atherosclerosis) by releasing several mediators with local and systemic effects (i.e., adipocytokines) (Figure 1).

The hypertrophic visceral adipose tissue of obese patients undergoes degenerative remodeling including hypoxia and macrophage infiltration, favoring the development of inflammation with direct effects on the quality and quantity of released adipocytokines. The reduction of atheroprotective adiponectin (43) and the increased levels of resistin facilitate lipolysis with direct effects on vessel health (44). As exercise was shown to reduce resistin levels (45, 46), such adipokine can be a further link between exercise and PCSK9 since resistin was reported to increase the expression of PCSK9 in hepatocyte thereby modulating LDLR levels and indirectly atherogenesis (47). Furthermore, activation of adiponectin receptors was shown to regulate PCSK9 expression in experimental model of atherosclerosis with direct impact on the disease burden (48). Also, leptin interferes with PCSK9 expression *via* the activation of STAT3 and p38MAPK pathways (49, 50). Among, adipocytokines, fibroblast growth factor 21 is a peptide implied in fatty acids and glucose metabolism (51) under both physiological and pathological conditions including obesity and metabolic syndrome (52). Acute exercise was shown to increase the circulating levels of fibroblast growth factor 21 (53), which in turn impairs the expression of PCSK9 *via* the suppression of the hepatic sterol regulatory element binding protein 2 (54). Expressed by a variety of cell types including adipocytes, annexin A2 is an anionic phospholipid-binding proteins of the Ca^2+^ dependent family that was reported to reduce PCSK9 levels by favoring the modification of its catalytic subunit (55–57). Of interest, annexin A2 levels are elevated in people who regularly practice exercise (25), thereby indicating another possible molecular link.

Recently described as an important mediator of atherogenesis, lectin-type oxidized low-density lipoprotein receptor-1(LOX-1) may be another mediator of the effect of PA on PCSK9 levels. LOX-1 is a scavenger receptor with important function in oxidized LDL-C uptake by endothelial cells (58). PCSK9 and LOX-1 are both involved in the atherosclerotic process (59) and recent evidence showed that PCSK9 and LOX-1 influence each other in the vascular tissue (18, 60) and are co-related to the atheroma formation. Pre-clinical evidence showed that PA can reduce cholesterol accumulation in atherosclerotic plaques *via* the reduction of LOX-1 gene expression (61). Both PCSK9 and LOX-1 are reduced upon exercise (22).

Sterol regulatory element binding protein 2 (SREBP2) is a molecule implicated in the synthesis of cholesterol (62) as well as a transcriptional activator of PCSK9 (63, 64). For such reason, SREBP2 is often used to explain the paradoxical association of elevated PCSK9 levels and reduced LDL-C that is seen during fasting periods.

Even though it is not clear whether PA can modulate SREBP2 levels, regular exercise reduces the expression of several enzymes that regulates lipid metabolism in animal models (65, 66). The reduction of SREBP2 levels would end in lower PCSK9 plasma levels, as it happens during fasting (67, 68). Indeed, few days of fasting reduce the amount of circulating PCSK9 but with the unexpected results of increasing the quantity of circulating LDL-C (69).

Furthermore, regular PA can augment the circulating levels of interferon gamma, a cytokine with critical role in regulation of immune system, as showed in both pre-clinical (70) and clinical (71, 72) settings. Recent studies suggested a role for interferon gamma in PCSK9 regulation (73).

An excess of body fat is related to several detrimental is associated with higher risk of insulin resistance (74), and insulin resistance is one of the key element of the metabolic syndrome. Also, higher levels of insulin are associated with a higher expression of PCSK9 *via* sterol regulatory element binding protein 1c (SREBP-1c) (17). On the other hand, regular physical activity can improve insulin sensitivity (75–77).

### Pitfalls in determining the effect of PA on PCSK9

First of all, the variations of LDL-C circulating levels can either be a consequence or a cause of PCSK9 variations. In fact, when PCSK9 modulate the amount of LDL-C also LDL-C can directly bind the PCSK9 molecule, causing a direct impairment of its functionality (78). Furthermore, many limitations reside in the evaluation of circulating levels of this mediator as it is not clear whether this is a fair counterpart of its hepatic levels. Also, as most studies investigate the levels of this molecule, they might not directly reflect its activity. Under this point of view, the presence of studies showing augmentation of PCSK9 along with reduction of LDL-C circulating levels might be explained by different PCSK9 functionality. Furthermore, most of the analytic method for detecting PCSK9 use antibodies binding to its mature form. However, the activity of PCSK9 resides in its catalytic processes (79).

As previously mentioned, another great limitation resides in the difficult standardization of exercise protocols. Also, with regard to clinical research, sample sizes are generally small, and some studies has non-negligible unbalanced gender and/or comorbidities differences. The interpretation of the relatively small number of recent clinical trials is also hampered by the fact that often they use holistic interventional program with prescription of both PA and diet. Lastly, the concomitant use of statin therapy can interfere with the interpretation of the results. In fact, statin treatment augments the amount of circulating PCSK9 (80–82), probably because they increase the levels of SREBP2 (83).

## Conclusions

Regular exercise is known to ameliorate lipid profile and represent the cornerstone of cardiovascular prevention. Patient that practice regular exercise show a non-negligible reduced risk of developing atherosclerosis and possible cardiovascular events. The relationship between PCSK9 and PA can be a possible explanation with exercise training envisaging a non-pharmacological PCSK9 inhibitor. Indeed, the reduction of adiposity and molecules like LOX-1, annexin A2, fibroblast growth factor 21, and resistin secondary to exercise favor the reduction of PCSK9 in the bloodstream. Yet, at present, several limitations impact on the interpretation of the results including the difficult standardization of exercise protocols.

## Author contributions

AT conceived and drafted the manuscript. FM and LL revised it for important intellectual content. All authors read and approved its final version.

## Funding

LL received a research grant from the Swiss Heart Foundation.

## Conflict of interest

Author LL is co-inventor on the International Patent WO/2020/226993 filed in April 2020; the patent relates to the use of antibodies which specifically bind interleukin-1α to reduce various sequelae of ischemia-reperfusion injury to the central nervous system. The remaining authors declare that the research was conducted in the absence of any commercial or financial relationships that could be construed as a potential conflict of interest.

## Publisher's note

All claims expressed in this article are solely those of the authors and do not necessarily represent those of their affiliated organizations, or those of the publisher, the editors and the reviewers. Any product that may be evaluated in this article, or claim that may be made by its manufacturer, is not guaranteed or endorsed by the publisher.
